# Triple artemisinin-based combination therapy (TACT): advancing malaria control and eradication efforts

**DOI:** 10.1186/s12936-024-04844-y

**Published:** 2024-01-18

**Authors:** Emmanuel Kokori, Gbolahan Olatunji, Adeola Akinboade, Aminat Akinoso, Emmanuel Egbunu, Sikiru Ademola Aremu, Chuka Emmanuel Okafor, Olamide Oluwole, Nicholas Aderinto

**Affiliations:** 1 Department of Medicine and Surgery, University of Ilorin, Ilorin, Kwara Nigeria; 2 Federal Medical Centre, Bida, Niger Nigeria; 3 Lagos University Teaching Hospital, Lagos, Nigeria; 4 Afe Babalola University, Ado Ekiti, Nigeria; 5 Department of Medicine, Ladoke Akintola University of Technology, Ogbomosho, Nigeria

**Keywords:** Triple artemisinin-based combination therapies, Malaria control, Malaria eradication, Drug resistance

## Abstract

This paper examines the far-reaching implications of Triple Artemisinin-Based Combination Therapy (TACT) in the global battle against malaria. Artemisinin-Based Combination Therapy (ACT) is recognized for its cost-effectiveness, lower likelihood of adverse events, and widespread acceptance by patients and healthcare providers. However, TACT introduces novel dimensions to the fight against malaria that make them a superior choice in several aspects. TACT has been demonstrated to address resistance, offer a broader spectrum of action, reduce the risk of treatment failure, and can be tailored to meet regional needs, strengthening the global effort to combat malaria. However, maximizing these benefits of TACT depends on accessibility, particularly in resource-limited regions where malaria is most prevalent. Collaborative efforts among stakeholders, sustainable pricing strategies, efficient supply chains, and public–private partnerships are essential to ensure that TACT reaches needy populations. Moreover, dispelling prevalent malaria myths through health education campaigns is critical in this endeavour. The paper underscores the significance of collaborative initiatives and partnerships among governments, international organizations, research institutions, acadaemia, pharmaceutical companies, and local communities. Together, these efforts can pave the way for the acceptance, adoption, and success of TACT, ultimately advancing the global goal of a malaria-free world.

## Background

Malaria persists as a formidable global health challenge, claiming countless lives despite extensive efforts in prevention and treatment. According to the World Health Organization (WHO), in 2021 alone, there were a staggering 247 million reported malaria cases worldwide, resulting in a tragic toll of 619,000 lives lost [1]. Shockingly, this disease remains a leading cause of mortality, with children under five particularly vulnerable [2]. While malaria's endemicity is most pronounced in the sub-Saharan African region, its insidious reach extends to the sparse remaining pockets in Southeast Asia [1]. The economic burden is immense, with an estimated global expenditure of $4.3 billion in 2016, and its adverse impact extends to national economies, where a 1.3% reduction in Gross Domestic Product (GDP) per person is observed in countries grappling with intense transmission [3].

Malaria's impact on human health dates back to 2700 BCE in China, with a history marked by identifying the malaria parasite (*Plasmodium* spp.) and its vector, the *Anopheles* mosquito, in the nineteenth century [4]. The twentieth century saw an estimated 150–300 million lives lost to malaria, comprising 3–5% of all global deaths [5]. Despite progress in the twenty-first century with malaria elimination in 27 countries, the disease remains a significant public health threat, affecting roughly half of the world’s population across 108 countries [4]. Historically, treatments for malaria included quinine, mepacrine, chloroquine, sulfadoxine-pyrimethamine, and mefloquine, each facing the challenge of resistance development [6–8]. The introduction of artemisinin in 1971 marked a breakthrough in malaria treatment, as it exhibited high efficacy against multi-drug resistant malaria [7]. 2005 marked a significant turning point in the battle against malaria with the introduction of artemisinin-based combination therapy (ACT), the combination of an artemisinin derivative and a partner drug with a longer action [4, 9]. However, isolated reports of increasing artemisinin resistance raised concerns, leading to the exploration of innovative solutions [4].

Triple artemisinin-based combination therapy (TACT) emerged as a novel approach to combat resistance to ACT. TACT merges an established artemisinin-based combination therapy with a long-lasting partner medication to combat resistance [9]. Ongoing research seeks to uncover the rationale and assess the pharmacogenetics and pharmacodynamics of triple artemisinin-based combinations, although current knowledge is limited [10]. Notably, combinations such as artemether-lumefantrine with amodiaquine, and dihydroartemisinin-piperaquine with mefloquine, combine a potent but short-acting artemisinin component with two longer-acting components, strategically combating drug resistance [9]. In the artemether-lumefantrine-amodiaquine combination, lumefantrine binds to haemin, preventing detoxification into haemozoin, while artemether releases toxic free radicals [11]. Amodiaquine complements this action by inhibiting protozoan DNA, RNA, and protein synthesis [12]. Dihydroartemisinin-piperaquine combines the rapid action of dihydroartemisinin (DHA) and the slow action of piperaquine (PPQ) for mass drug administration (MDA) [7]. DHA produces radical oxygen species post-hem activation, leading to parasite cell damage, while PPQ disrupts metabolic processes by inhibiting haemozoin formation [12]. Adding mefloquine enhances the efficacy of DHA-PPQ and reduces resistance to piperaquine [9]. Each drug matches the pharmacokinetics of the partner drugs. For artemether-lumefantrine-amodiaquine, the three anti-malarials exert an inverse selection pressure on the Pfmdr1 N86Y allele [11]. For artesunate mefloquine-piperaquine amplification of *Pfmdr1* and *PfPlasmepsin2/3* in the same parasites appears extremely rare [9]. In light of the evolving landscape of malaria treatment and the recent emergence of artemisinin partial resistance, initially identified on the Thai-Cambodian border in 2008 and now documented in sub-Saharan Africa, the global malaria research community has been diligently exploring various mechanisms to counteract the effects of this resistance [13]. These developments place additional pressure on ACT which is widely used as first-line treatment in African countries. In response to these challenges, mechanisms such as adopting multiple first-line therapies and the early institution of TACT have been actively advocated. This paper explores the potential of TACT in shaping the future of malaria control and prevention strategies. It examines the rationale, effectiveness, challenges, and prospects of TACT.

### Current evidence on TACT

#### Efficacy of TACT

One pivotal clinical trial of TACT is the TRACII trial, conducted by the Tracking Resistance to Artemisinin Collaboration. This multinational trial spanned 18 sites across eight countries, enrolling patients aged 2–65 with acute, uncomplicated *Plasmodium falciparum* malaria. Participants were randomly assigned to one of two treatments based on location, with the primary outcome assessed as the PCR-adjusted adequate clinical and parasitological response at day 42 [9]. The results from the TRACII trial demonstrated that TACT was non-inferior to ACT in terms of efficacy and displayed similar safety profiles. Furthermore, TACT exhibited a reduced risk of *P. falciparum* reinfection and delayed time to reinfection compared to ACT. This trial’s findings suggest that TACT holds promise in extending the therapeutic lifespan of existing anti-malarials and curbing the spread of drug resistance [9].

Another valuable tool in the assessment of TACT is the TACTS module, a software tool designed for simulating data from various types of trials and summarizing the results. Users can customize trial parameters, including sample size, treatment arms, outcome measures, effect sizes, and randomization methods. The TACTS module generates simulated data sets, reflecting the trial design, and provides summary statistics and graphical representations of outcomes. This tool explores the feasibility, power, sensitivity, and robustness of different TACTs trial designs [14].

A comparative analysis between TACT and traditional ACT is essential in malaria treatment. TACT represents a novel approach to overcoming the resistance that *P. falciparum* has developed against conventional ACT, which typically employ only two active components [15]. A recent study by the Mahidol Oxford Tropical Medicine Research Unit (MORU) shed light on the effectiveness of TACT, particularly in regions like Cambodia, Vietnam, and Thailand, where traditional ACT faces substantial challenges due to multidrug resistance [9]. In these areas, triple artemisinin-based combinations have shown great promise as a potential solution. The study compared TACT using dihydroartemisinin-piperaquine plus mefloquine, or artemether-lumefantrine plus amodiaquine with the standard ACT dihydroartemisinin-piperaquine (DHA-PPQ) and revealed that all triple artemisinin-based combinations achieved cure rates exceeding 95%, while the cure rates of DHA-PPQ remained below 50%. Additionally, TACTs reduce the risk of reinfection and transmission of the parasite [16]. However, it is essential to acknowledge that TACT, while effective, may not serve as a long-term solution, as resistance can eventually develop even against these innovative therapies. As such, the study authors recommended deploying TACT in conjunction with complementary strategies, such as enhanced surveillance, vector control measures, and developing new anti-malarial drugs and vaccines [14].

#### Safety and tolerability

Two triple artemisinin-based combinations that have been tested have demonstrated overall safety and good tolerability. Incidences of vomiting during the first hour of treatment with both triple artemisinin-based combinations were low. However, it is worth noting that adding mefloquine or amodiaquine to existing artemisinin-based combinations was associated with a slight increase in the incidence of vomiting [17]. Furthermore, adding amodiaquine slightly prolonged the QTc interval (a measure of the heart’s electrical activity), although it did not reach levels associated with cardiac arrhythmias. Importantly, both triple artemisinin-based combinations exhibited high efficacy, particularly in Asian regions. While TACT was generally safe and well-tolerated, some adverse effects, such as loss of appetite, nausea, and vomiting, occurred slightly more frequently than standard treatments. Nevertheless, these findings suggest that TACT holds promise as effective options for treating drug-resistant malaria.

A study conducted across geopolitical zones in Nigeria observed several common adverse effects of anti-malarial treatments, including general body weakness, dizziness, vomiting, abdominal pain, insomnia, body pains, and anorexia [18]. These common side effects of anti-malarial drugs often overlap with symptoms of malaria infection, potentially leading to an overestimation of anti-malarial side effects [9]. Combining an artemisinin-based combination with another drug may result in additional side effects or intensify those associated with anti-malarial treatment. Quantifying the increased risk of adverse events and determining whether they are dose- or concentration-dependent is essential. While several current anti-malarials, including quinine, chloroquine, amodiaquine, mefloquine, and piperaquine, have cardiovascular side effects, they remain within acceptable safety limits when used at therapeutic doses [5]. Adding amodiaquine to artemether-lumefantrine slightly increased the prolongation of the QTc interval, comparable to the administration of amodiaquine alone, without raising significant safety concerns [19, 20]. Other less common side effects include tinnitus, bradycardia, type 1 hypersensitivity reactions, transient reticulocytopenia, neutropenia, and elevated liver enzymes [21].

One strategy to minimize side effects and improve patient adherence involves combining anti-malarial drugs with minimal interactions to reduce expected side effects. Quantifying the increased risk of adverse events and assessing whether they are dose or concentration-dependent can aid in formulating preferred dosage recommendations for each combination [22]. Efforts must be made to educate patients and healthcare staff at all levels about the advantages and rationale for using TACT instead of ACT. Clear communication is crucial to ensuring the successful adoption of TACT [23]. While it is anticipated that triple artemisinin-based combinations may be more expensive per treatment course than conventional artemisinin-based combinations, governments and global organizations, such as the Global Fund, could consider subsidizing the cost to make TACT more affordable and widely accessible, thereby improving patient adherence [24].

#### Global impact and accessibility

TACT represents a promising advance in the global fight against malaria, offering new avenues for worldwide malaria control efforts. By delaying the development of drug resistance in artemisinin-based treatments, TACT may prevent the regression of progress in malaria control. This delay allows the discovery of alternative therapies and preventive measures, potentially paving the way for complete eradication in some regions.

Adopting TACT can catalyse healthcare access and infrastructure improvements in malaria-endemic areas. Despite the potential benefits of TACT, their viability hinges on accessibility, particularly in resource-limited areas where malaria is most prevalent. Collaboration among stakeholders is vital to ensure that TACT reach needy populations. Pricing strategies must be carefully examined to make TACT affordable. Cooperation between pharmaceutical companies, international organizations, and governments is essential. Mechanisms such as subsidies and donor support can play a crucial role in maintaining reasonable prices.

Efficient and reliable supply chains are crucial for delivering TACT to isolated and underserved regions. Developing and implementing distribution and procurement strategies that minimize supply chain disruptions is imperative. Many underserved areas lack robust insurance plans, leading most individuals to pay out of pocket. Collaboration between the public and private sectors can streamline TACT production, distribution, and accessibility. Such partnerships can potentially lower the cost of TACT. Additionally, engaging with local communities ensures acceptance and proper usage. Tailored education and awareness programmes can debunk common myths and build trust in the healthcare system. All these efforts underscore the importance of collaboration among diverse stakeholders. Governments, international organisations, and research institutions must coordinate efforts, share knowledge, and allocate resources effectively. Initiatives like the Global Fund and the Roll Back Malaria Partnership facilitate such collaboration.

Collaborations involving academia, pharmaceutical companies, research organisations, local communities, non-governmental organisations (NGOs), and community health workers should unite with a shared purpose. Their primary goal should be to accelerate the process of discovering more suitable combinations and formulations. In addition to research, these collaborations should enhance their understanding of the local contexts where TACT will be deployed. Local communities, NGOs, and community health workers are essential stakeholders whose active participation is key to the success of malaria control initiatives. Their insights into the unique challenges and needs of their regions are invaluable. Collaborating with them ensures that TACT promotion and deployment strategies are finely tuned to the specific requirements of each locality.

#### Challenges and future directions

**Fig. 1:**
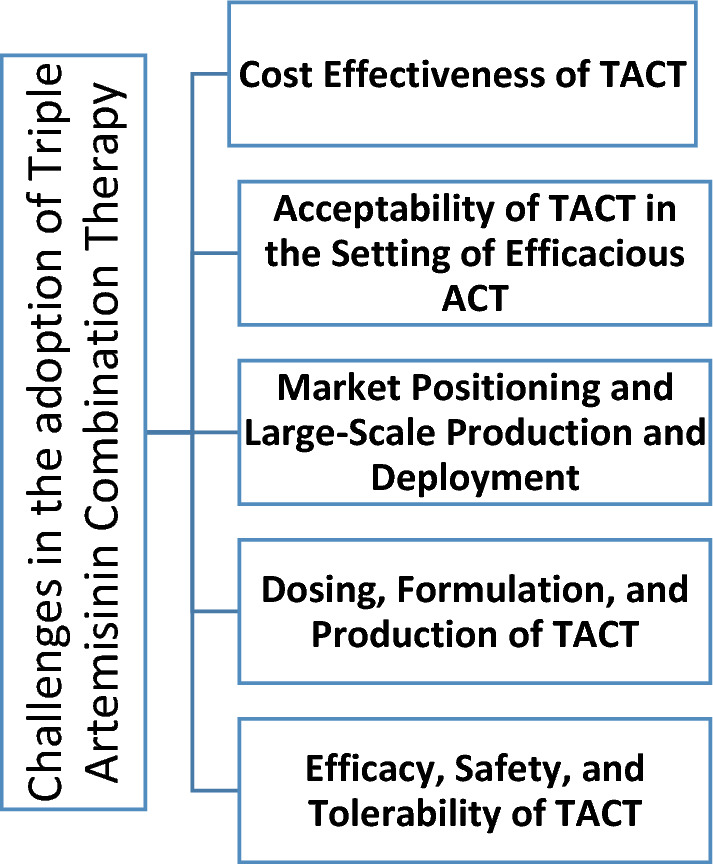
Challenges in the adoption of Triple Artemisinin-Based Combination Therapy

The widespread adoption of ACT faced several obstacles, including cost, availability, decision-making delays, and concerns among policymakers, healthcare providers, and patients. Similar challenges and additional ones are likely to emerge in the potential rollout of TACT. See Fig. 1.

#### Dosing, formulation, and production of TACT

Optimizing TACT composition and dosing regimens necessitate considering age-stratified pharmacokinetic drug profiles, dose–effect relationships, and dose-related toxicity and tolerability [25, 26]. Inadequate dosing of any TACT component can lead to incomplete parasite clearance, subsequent recrudescences, and the selection of drug-resistant parasites [27, 28]. While coformulation ensures accurate dosing, pharmaceutical issues occasionally affect drug stability. Additionally, assessing in vivo interactions among the combination drugs is crucial [29]. Various factors will determine the additional costs associated with TACT development. However, most currently used anti-malarials are off-patent, limiting the costs of individual drugs [30]. While coformulation is preferable for patient adherence, an ACT and a second partner drug could be considered a more rapidly available option.

#### Efficacy, safety, and tolerability of TACT

The safety and tolerability of all presently available artemisinin-based combinations and individual anti-malarial components are well-established. Common side effects of anti-malarials drugs include fatigue, headache, dizziness, nausea, vomiting, and abdominal pain [30]. These symptoms often overlap with those of malaria infections, leading to overestimating antimalarial side effects. It is vital to quantify the increased risk of adverse events and determine whether they are dose- or concentration-dependent. The TRACII trial (with 1100 patients randomised to receive various treatments) showed that adding mefloquine to DHA–PPQ or amodiaquine to AL did not raise significant safety concerns regarding QTc-interval prolongation [31].

#### Cost effectiveness of TACTs to prevent or delay the emergence of resistance

Studies on TACT have not fully addressed the potential long-term benefits of deploying TACT to prevent or delay the development of anti-malarial drug resistance. Mathematical modelling has been instrumental in identifying the role of mismatched pharmacokinetic and pharmacodynamic (PK/PD) profiles in promoting the evolution of drug-resistant malaria [32]. Recent developments include PK/PD modelling to identify suitable candidates for development, incorporating laboratory measures of drug resistance and resistance spread, and transmission models to guide drug deployment scenarios [33, 34]. Mathematical modelling has estimated that ACT failure could lead to a 28% increase in direct medical costs for malaria treatment (US$ 146 million) and a US$ 130 million cost of policy change if switching between different artemisinin-based combinations were necessary [35]. Extensions of these models can provide insights into the future impact of deploying novel antimalarial therapies by acknowledging inherent model limitations and assumptions. Mathematical modelling can assess the likely epidemiological impact of TACT as a first-line anti-malarial treatment in diverse malaria-endemic settings. This quantification can help determine the potential of TACT to delay the emergence and spread of artemisinin- and partner-drug-resistant falciparum malaria under different transmission intensities, population mobility, and fitness costs of drug resistance mutations. Pharmacokinetic modelling for partner drugs can determine the dose at which toxicity is minimized while maintaining sufficiently high drug levels to eliminate parasites not cleared in the initial 3 days of treatment, thereby shortening the window of selection during which parasites are exposed to suboptimal drug levels [36]. Economic modelling can assess the cost-effectiveness of deploying TACT in various endemic settings, factoring in potential additional costs versus savings from reducing malaria morbidity and mortality due to anti-malarial drug resistance.

#### Acceptability of TACT in the setting of efficacious ACT

Ethical considerations in malaria control and elimination require attention to minimize potential infringements on individual autonomy while ensuring a favourable risk–benefit balance [37]. In the absence of widespread ACT failure, introducing TACT as the first-line treatment in Africa raises ethical questions. These questions parallel the ethical considerations surrounding mass anti-malarial drug administration, where long-term community benefits may not be immediately guaranteed [38]. In Africa, social and cultural factors previously delayed the adoption of ACT over conventional, less effective anti-malarials [39]. Therefore, prior community engagement is essential to devise culturally sensitive methods that promote acceptance and adherence to TACT. Thoroughly examining stakeholders' perspectives, ranging from patients and parents to regulatory authorities and national malaria control programs, will contribute to developing recommendations for TACT deployment in Africa. Without such engagement, TACT acceptance may be low, diminishing their impact in preventing or delaying emergent resistance. The recent emergence of artemisinin resistance in Rwanda may alter TACT deployment's risk–benefit and ethical analyses in Africa, potentially making TACT more urgently needed.

#### Market positioning and large-scale production and deployment of TACT

Transitioning to ACT for malaria treatment was a significant challenge in recent malaria control efforts [40]. Cost was a key factor preventing governments from switching to ACT [41]. Beyond supply and demand dynamics, market instability hindered widespread ACT adoption. Coordination issues in distribution chains and private sector compliance posed additional challenges. Various stakeholders play roles in deploying new antimalarials, and their views, attitudes, and concerns must be assessed early to develop strategies for appropriate TACT market positioning. Manufacturers need convincing that investing in TACT development and production is justified. While a substantial market exists (with approximately 3 billion ACT treatment courses procured worldwide between 2010 and 2018), malaria primarily affects underprivileged and poor populations. Therefore, anti-malarial drug prices must remain highly affordable for both the private and public sectors. Dialogue between manufacturers and stakeholders, including WHO, national malaria-control programmes, academic groups, civil society representation, Medicines for Malaria Venture (MMV), and funding organizations like the Global Fund, Wellcome, Bill and Melinda Gates Foundation, is crucial to support manufacturers in their decision-making process.

#### Role of health education and community engagement

Health education plays a pivotal role in garnering acceptance and adoption of TACT in the fight against malaria. The emergence and spread of anti-malarial drug resistance, particularly to artemisinins, pose significant threats to global malaria control and elimination efforts. Health education campaigns should prioritise raising awareness about TACT, disseminating scientific findings, debunking myths, and providing targeted healthcare provider training. Addressing prevalent misconceptions is crucial in the battle against malaria. One pervasive myth suggests that the absence of mosquito buzzing implies safety, which is debunked by the fact that female *Anopheles* mosquitoes possess near-silent flight mechanisms. Additionally, the myth that certain seasons confer safety from malaria-carrying mosquitoes is inaccurate, as malaria transmission can persist year-round. Comprehensive protection from malaria necessitates supplementary measures, including bed nets and prevention, especially in high-endemicity regions. Fostering accurate information, evidence-based interventions, and continued scientific research are essential to malaria control and elimination efforts.

## Conclusion

TACT represents a beacon of hope in the ongoing fight against malaria, offering substantial potential to reduce the global burden of this devastating disease. The comprehensive strategy of TACT promises improved treatment efficacy, accelerated parasite clearance, and a potential delay in developing drug resistance. These benefits translate into saved lives, reduced suffering, and strengthened public health systems, particularly in malaria-endemic regions. However, the realization of TACT potential hinges on their accessibility. Ensuring these therapies reach the populations most in need demands collaborative efforts from various stakeholders. Sustainable pricing, efficient supply chains, and public–private partnerships are crucial components of this accessibility equation. Moreover, health education campaigns aimed at dispelling common malaria myths are essential in promoting the acceptance and adoption of TACTs, emphasizing the importance of evidence-based interventions and accurate information. Collaboration among governments, international organizations, research institutions, acadaemia, pharmaceutical companies, and local communities is paramount. Initiatives such as the Global Fund and the Roll Back Malaria Partnership are pivotal in fostering these partnerships and accelerating malaria control and elimination progress.

## Data Availability

Data sharing is not applicable to this article as no datasets were generated or analysed during the current study.

## Abbreviations

TACTs: Triple Artemisinin-Based Combination Therapies
WHO: World Health Organization
GDP: Gross domestic product
ACT: Artemisinin-based combination therapy
CDC: Centers for disease control and prevention
MDA: Mass drug administration
MORU: Mahidol oxford tropical medicine research unit
